# Mechanisms of hyperprogressive disease after immune checkpoint inhibitor therapy: what we (don’t) know

**DOI:** 10.1186/s13046-020-01721-9

**Published:** 2020-11-09

**Authors:** Simone Camelliti, Valentino Le Noci, Francesca Bianchi, Claudia Moscheni, Francesca Arnaboldi, Nicoletta Gagliano, Andrea Balsari, Marina Chiara Garassino, Elda Tagliabue, Lucia Sfondrini, Michele Sommariva

**Affiliations:** 1 Dipartimento di Scienze Biomediche per la Salute, Università degli Studi di Milano, via Mangiagalli 31, 20133 Milan, Italy; 2 Molecular Targets Unit, Department of Research, Fondazione IRCCS – Istituto Nazionale dei Tumori, via Amadeo 42, 20133 Milan, Italy; 3 Thoracic Oncology Unit, Medical Oncology Department, Fondazione IRCCS Istituto Nazionale dei Tumori, via Venezian 1, 20133 Milan, Italy

**Keywords:** Hyperprogressive disease, Immune checkpoint inhibitors, Immunotherapy, Immune-mediated mechanisms

## Abstract

Immune checkpoint inhibitors (ICIs) have made a breakthrough in the treatment of different types of tumors, leading to improvement in survival, even in patients with advanced cancers. Despite the good clinical results, a certain percentage of patients do not respond to this kind of immunotherapy. In addition, in a fraction of nonresponder patients, which can vary from 4 to 29% according to different studies, a paradoxical boost in tumor growth after ICI administration was observed: a completely unpredictable novel pattern of cancer progression defined as hyperprogressive disease. Since this clinical phenomenon has only been recently described, a universally accepted clinical definition is lacking, and major efforts have been made to uncover the biological bases underlying hyperprogressive disease. The lines of research pursued so far have focused their attention on the study of the immune tumor microenvironment or on the analysis of intrinsic genomic characteristics of cancer cells producing data that allowed us to formulate several hypotheses to explain this detrimental effect related to ICI therapy. The aim of this review is to summarize the most important works that, to date, provide important insights that are useful in understanding the mechanistic causes of hyperprogressive disease.

## Background

### Immune checkpoints and immune checkpoint blockade: a brief overview

Immune checkpoints (ICs), such as cytotoxic T lymphocyte-associated protein 4 (CTLA-4), programmed cell death protein 1 (PD-1) and programmed cell death ligand 1 and 2 (PD-L1 and –L2), belong to complex regulation systems that are physiologically involved in fine tuning the immune response and in the prevention of autoimmune reactions [1]. However, the same mechanisms can be exploited by cancer cells to elude immune system attack [2]. Indeed, it is well known that the upregulation of these molecules in immune cells infiltrating the tumor microenvironment (TME) or on cancer cells themselves can lead to immune escape and, therefore, to poor prognosis [3]. Blockade of ICs by specific antibodies (immune checkpoint inhibitors, ICIs) determines a sort of “awakening” of antitumor immunity and represents an excellent and novel therapeutic strategy in the oncology field [4]. Several clinical studies have demonstrated the efficacy of these therapeutic approaches compared with conventional therapies. Response rates following ICI therapy (anti-CTLA4 or anti-PD-1/PD-L1 antibody therapy) were observed in 13.3 to 44% of patients with head and neck squamous cell carcinoma (HNSCC) [5], advanced melanoma [6–11], non-small cell lung cancer [12–14], renal cell carcinoma [15, 16], Merkel cell carcinoma [17] and metastatic urothelial carcinoma [18]. Notably, a recently published retrospective cross-sectional study, performed considering the highest response rate to ICI treatment for all tumor types, estimated that in 2018, the percentage of US cancer patients who received benefit from IC blockade was 12.46% [19]. These data indicate that most patients do not respond properly to therapy and progress despite treatment. The reasons for such a lack of benefit can be ascribed to different causes that can be grouped into two broad areas, defined as primary and acquired resistance mechanisms [20, 21]. The former, where certain characteristics of cancer cells/TME prevent ICIs from being efficacious and, therefore, patients do not respond to immunotherapy, can be determined by the lack of tumor antigenic mutations or defective antigen presentation, constitutive PD-L1 expression, impaired T cell tumor infiltration and function and an increased presence of immune suppressive cells within the tumor microenvironment TME [20, 21]. The latter, which occurs in patients who, after exhibiting an initial response to immunotherapy, eventually relapse and progress, includes phenomena such as aberrant activation of Janus kinases 1 and 2 (JAK1/2), leading to impaired sensitivity to IFN-γ produced by activated T cells and upregulation of alternative coinhibitory immune checkpoints [20, 21].

### Hyperprogressive disease (HPD): an unexpected outcome to ICI therapy

Moreover, among the nonresponders, it is possible to identify a population of patients, whose percentage can vary from 4 to 29% according to different studies [22], who experience an extremely rapid increase in tumor growth and metastatic spread after ICI administration, a dramatic progression of disease that, for this reason, was called hyperprogressive disease (HPD). It is worth mentioning that HPD represents a completely different pattern of progression and is not superimposable with conventional progressive disease or pseudoprogression. One of the first clues about the existence of HDP was provided by the observations made by Chubachi et al., who described a patient with non-small cell lung cancer (NSCLC) characterized by an acceleration in tumor growth during anti-PD-1 therapy, a phenomenon that the authors named “disease flare” [23]. In 2017, the work by Champiat et al. provided the first systematic description and a clear definition of HPD [24]. According to RECIST1.1 criteria, they classified HPD as those tumor progressions with a minimum two-fold increase in tumor growth rate (TGR) after initiation of therapy compared to the pretherapy TGR [24]. In this study, 218 patients with different types of cancers were treated with anti-PD1 or anti-PD-L1 antibody monotherapy. Among 131 patients evaluable for the analysis, 12 of them, representing 24% of patients diagnosed with disease progression, were classified as having HPD. Moreover, survival analysis demonstrated that compared to patients with complete or partial response, HPD patients were characterized by a 25.94-fold increase in dying. Another important finding was that HPD was not restricted to a specific tumor histotype but involved a broad spectrum of cancers. The pioneering publication of Champiat et al. was then supported and corroborated by other clinical studies [25–31] (Table 1).

**Table 1:** HPD rates in different clinical studies

Type of cancer	N° of patients	HPD rate (%)	Reference
Different types of cancer	131	9	[24]
NSCLC	335	13.1	[25]
Gastric cancer	36	11.1	[26]
Different types of cancer	182	6.6	[27]
NSCLC	406	13.8	[28]
NSCLC	152	25.7	[29]
HNSCC	34	29	[30]
Different types of cancer	155	4	[31]

Since HPD was only recently brought to the attention of the scientific community, there are many aspects that need to be clarified and that still represent a matter of debate. First, there are no internationally accepted guidelines to identify HPD patients, and every single research group utilizes its own criteria based on radiological or clinical parameters. For example, the criteria described in [24] were made more stringent in a subsequent paper published by the same research group. Indeed, the authors defined HPD as a RECIST 1.1 progressive disease at first evaluation with a ∆TGR (difference between TGR on therapy and TGR pretherapy) greater than 50% [28]. Another example of radiological definition of HPD was provided by Bouzid et al., who considered the tumor growth kinetics (TGK), defined as the difference in the sum of the largest diameters of target lesions (according to RECIST criteria) per unit of time. A TGK_R_ ≥ 2 (= the ratio between TGK on therapy and TGK before therapy) was an indicator of HPD [30]. One of the main disadvantages of criteria based on radiological evaluations is that prebaseline computed tomography (CT) scans are not always available, especially in the case of first-line therapy. Therefore, other studies provided definitions of HPD also considering clinical parameters. For instance, in their classification of HPD, Kato et al. also considered time-to-treatment failure (TTF), defined as the time from the start of treatment to discontinuation for any reason. In their work, they described HPD as a condition with a TTF lower than 2 months, an increase of more than 50% in tumor burden compared to pretherapy imaging and a more than two-fold increase in progression pace [31]. Moreover, in the study by Lo Russo et al., patients were diagnosed with HPD when they showed at least three of these criteria: TTF less than 2 months, an increase of more than 50% of the sum of target lesion major diameters between baseline and first radiological evaluation, appearance of at least two new lesions in an organ already involved based on baseline and first radiological evaluation, spread of the disease to a new organ, and a decrease in Eastern Cooperative Oncology Group (ECOG) performance status more than 2 during the first 2 months of treatment [29]. Considering the existence of several alternative definitions of HPD, summarized by Frelaut et al. [32], it follows that the percentage of HPD patients can vary widely among the different studies (from 4 to 29% of ICI-treated patients). Overcoming these differences is necessary to facilitate the interpretation of data obtained from different clinical studies and to begin rigorous epidemiological analyses in this field and, not least, to identify possible predictive markers of HPD that, to date, are not available. The discovery of possible predictive markers follows two different paths: the study of the host or the tumor [32, 33]. The former is focused on the patient’s general characteristics, such as age, gender, previous exposure to other therapies, or on the host immune system, such as the presence of a specific tumor-infiltrating or circulating immune cell population. The latter is aimed at analyzing intrinsic tumor features, such as specific genetic alterations, chromosomal instability, and tumor mutational burden.

Second, to date, the molecular and cellular mechanisms underlying the occurrence of HPD are not known. Since 2017, many hypotheses and speculations have been made to provide a fully convincing explanation of this extremely negative effect associated with ICI therapy.

### Hyperprogressive disease: an unsolved problem

In summary, HPD represents a serious problem in the era of immunotherapy for different reasons: (i) it involves a broad spectrum of cancers and is not restricted to a particular tumor histotype; (ii) there is no univocal definition of HPD; (iii) no reliable predictor markers have been identified yet; and (iv) the cellular and molecular mechanisms are still unclear despite several studies providing interesting suggestions and hypotheses. The aim of the present work is to provide an overview of the data available in the literature that might help to elucidate this clinical phenomenon. We also included some reports that, although not strictly related to HPD, allow us to understand that ICIs can trigger undesired effects.

## Possible immune-related mechanisms of HPD

PD-1 is an inhibitory receptor expressed on adaptive and innate immune cells such as T lymphocytes, B lymphocytes, monocytes, macrophages, natural killer (NK) cells, and dendritic cells (DCs) [34]. Upon engagement with its ligands (PD-L1 and -L2), this receptor triggers a signaling pathway leading to the inhibition of immune cell function, which can be reverted using specific antibodies able to disrupt the PD-1/PD-L1 axis [35]. ICIs enhance endogenous antitumor immune responses in patients with a wide range of cancers [4]. If the final outcome of ICI-based therapy strictly relies on how immune cells respond to anti-PD-1/PD-L1 antibodies, it is plausible to speculate that HPD may be caused by an undesired response of the immune system to these antibodies. It is difficult to address this question since PD-1 blockade is usually associated with the restoration of the immune response. However, it is possible to find some data that suggest that blocking PD-1 does not always attain the desired effect.

### Undesired effects of PD-1 blockade on innate immunity

NK cells actively participate in immune attack against tumors, but their cytotoxic activity is often dampened by the expression of immune checkpoints such as PD-1 [36]. PD-1 blockade not only elicits strong T cells but also restores the NK cell antitumor response [37, 38]. However, using a mouse model of gram-negative infection, Solaymani-Mohammadi et al. observed that PD-1 knock-out (KO) mice or anti-PD-1 antibody-treated mice were more susceptible to bacterial infection than wild-type (WT) mice. This observation was explained by the fact that the lack of PD-1 impairs the expression of NK cell effector molecules such as granzyme B and perforin [39]. Therefore, this work suggests that, in certain contexts, intact PD-1 signaling in NK cells is required to maintain the full functionality of these immune cells.

ILC1, ILC2 and ILC3 represent the three main subsets of helper innate lymphoid cells (ILCs) that can be considered the innate counterparts of CD4^+^ T helper 1 (T_H_1), 2 (T_H_2), and 17 (T_H_17) cells, respectively. ILCs contribute to the regulation of both the innate and adaptive immune systems through cytokine secretion after activation by different stimuli [40]. ILCs participate in the antitumor immune response or promote tumor development depending on the secreted cytokines and the tumor microenvironment in which they exert their immunomodulatory functions [41]. **A**nalyzing two patients who received anti-PD-1 immunotherapy and developed HPD after treatment, Xiong et al. found that ILC3-associated genes were enriched in tumors after anti-PD-1 administration, suggesting that the ILC3 population may be involved in HPD development [42]. However, the mechanisms by which ILC3 cells may promote HPD are still unknown. ILC3 cells have been reported to express functional PD-1 [43, 44], which plays a negative regulatory role in cytokine production upon PD-L1 engagement [43]. It is possible that the disruption of the PD-1/PD-L1 axis mediated by anti-PD-1 antibody might determine a reactivation of these cells with a consequent release of cytokines that, as already mentioned, can have protumor activity in certain types of cancers [45, 46]. Moreover, ILC3 can dampen the T cell response by competing with T cells for IL-2 [47], further strengthening the immunosuppressive microenvironment.

Dendritic cells (DCs) are the most potent antigen-presenting cells (APCs) able to induce effective adaptive immune responses and, for this reason, represent a key factor in determining the success of ICI therapy [48]. Tumor-infiltrating dendritic cells (TIDCs) are a heterogeneous myeloid immune population characterized by different maturation statuses and functions: some subsets have immunostimulatory properties able to promote antitumor immunity, while other subsets are immunosuppressive and can favor tumor immune escape and progression [49, 50]. As mentioned, PD-1 can also be expressed by DCs, and the presence in the tumor microenvironment of PD-1^+^ TIDCs has been observed [51]. PD-1 maintains DCs in an immature phenotype, suppressing cytokine production, costimulatory molecule expression and antigen presentation capacity [51, 52]. PD-1 expression can be regulated by different stimuli, such as IL-10 [53]. Interestingly, it was found that PD-1 blockade on PD-1^+^ DCs promotes an augmented release of IL-10 by these immune cells that, in turn, determines an upregulation of PD-1 on the DC cell membrane, creating a vicious loop eventually leading to enhanced immunosuppression [53]. Another intriguing function of PD-1 in DCs has been described by the work of Zhao et al. [54]. The authors demonstrated that PD-1 and its ligand PD-L1 are simultaneously coexpressed on a subset of tumor-infiltrating APCs that interact with each other on the same cell membrane. In this way, PD-1 on DCs sequesters PD-L1, neutralizing its ability to bind PD-1 expressed by T cells. After anti-PD-1 antibody administration, this binding is disrupted, and PD-L1 is again available to elicit immunosuppressive activity on T cells [54].

Monocytes are a population of innate immune cells that circulate in the bloodstream and move to tissues to reach the site of infection or inflammation. During cancer development, different monocyte subsets were found to have completely different functions contributing to both pro- and antitumor immunity. Moreover, monocytes are the primary source of tumor-associated macrophages (TAMs) and dendritic cells (DCs), two important components of the tumor microenvironment [55]. Although much less investigated than in T lymphocytes, PD-1 can also be expressed by monocytes, and its upregulation can be mediated by different inflammatory stimuli [56–59], such as IL-10, already described as a PD-1 inducer in DCs [53, 60]. The role of PD-1 in monocytes appears to be very similar to that described for other immune cells, a substantial inhibition of immune function. Indeed, PD-1 was reported to negatively impact IL-12 production by monocytes [61, 62], and PD-1 blockade reverses monocyte dysfunction [63]. However, monocytes release large amounts of IL-10 upon PD-1 triggering by specific antibodies [56, 64].

Although these data do not represent a mechanistic explanation of HPD, they allow us to understand that PD-1 biology is far from completely elucidated, especially in non-T cells. What emerges from these works is that, in specific cases, anti-PD-1 antibodies can exacerbate the immunosuppressive capacity of the cells of the innate immune system. This occurs, for example, during viral or bacterial infections or, sometimes, in tumors, all clinical conditions characterized by the high concentration and circulation of immunomodulatory molecules, such as cytokines, pathogen- or damage-associated molecular patterns (PAMPs or DAMPs) [65–67]. Innate immune cells are extremely adaptable, as they have the ability to completely change their phenotype and behavior as a result of the combination of the received stimuli [68]. These profound modifications may also have an impact on the pathways governed by PD-1. Therefore, PD-1 blockade might result in diametrically opposite outcomes depending on the phenotype acquired by these cells. A summary of the proposed innate immune-mediated mechanisms is provided in Table 2.

**Table 2:**
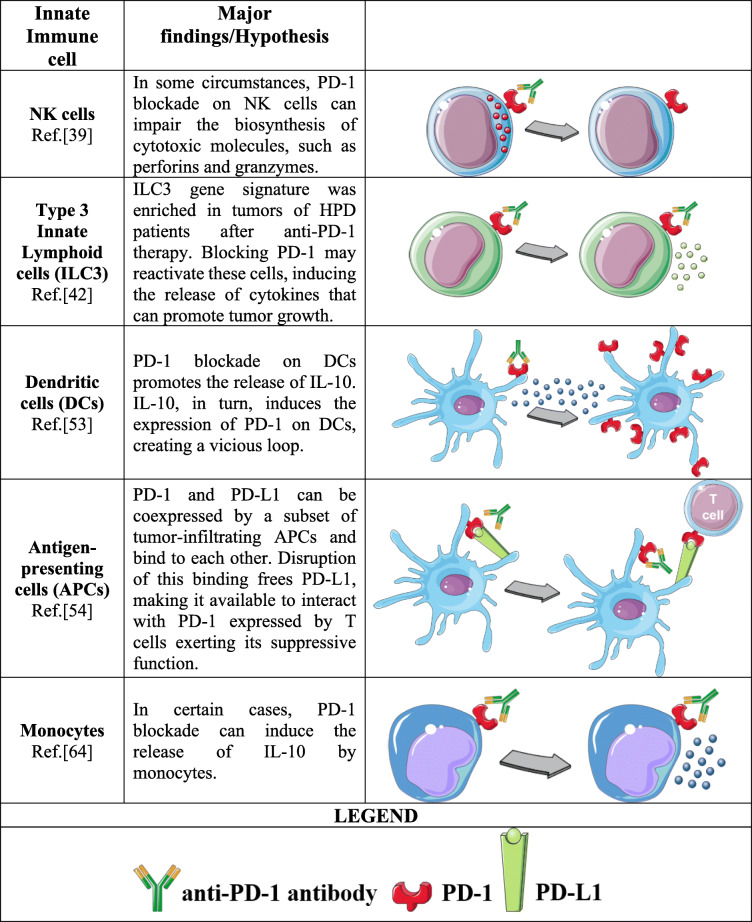
Possible immune-mediated mechanisms of HPD involving innate immune cells

Possible role of the innate immune system in determining HPD. In certain contexts, blocking PD-1 on innate immune cells may induce or exacerbate the immunosuppressive activity of these cells

### Unexpected effects of PD-1 blockade on adaptive immunity

Anti-PD-1 therapy was initially designed to restore T cell-based immune attack against tumors [35]. However, it is important to recall that PD-1 represents a master negative regulator of T lymphocyte function; therefore, its blockade might trigger compensatory mechanisms aimed at keeping T cell activity under control [69–71]. This negative regulatory feedback might overcome the benefit of anti-PD-1-based therapy in terms of T cell activation and might establish an enhanced immunosuppressive environment, creating the conditions for HPD development. For example, it has been reported that tumor-associated lymphocytes (TALs) or tumor-infiltrating lymphocytes (TILs), isolated from a mouse model of metastatic ovarian cancer or human ovarian cancer patients, simultaneously express several immune checkpoint inhibitory receptors, such as PD-1, LAG-3, and CTLA-4, that participate in creating an extremely immune-suppressed TME. Interestingly, the individual blockade of one of those receptors can lead to the up-regulation of the others, nullifying the benefit of ICI therapy [69]. On the same line, Koyama et al. observed, in mice and humans, an increased expression of the immune checkpoint T-cell immunoglobulin mucin-3 (TIM-3) on T cells after anti-PD-1 antibody administration [72]. However, these negative regulatory mechanisms are not only restricted to the immune checkpoints, but also involve other proteins that can contribute to the immune regulation. For instance, it was found that PD-1^high^ melanoma antigen-specific CD8^+^ T cells express high levels of IL-10 receptor (IL-10R) that can be further increased by PD-1/PD-L1 axis disruption, making these cells more sensitive to IL-10 [73]. If PD-1 blockade might induce IL-10 secretion by innate immune cells, as described in the previous section, and a parallel upregulation of IL-10R on T cells, these two combined events might cause severe dampening of CD8^+^ T cell antitumor activity, favoring tumor growth.

Moreover, the T lymphocyte family comprises not only effector cells but also an immunoregulatory subset, named regulatory T cells (T_regs_), whose duty is to negatively control other immune cells and to prevent autoimmunity [74]. In tumors, the presence of T_regs_ represents an unfavorable factor for the host since they constrain an efficacious antitumor immune response [74]. T_regs_ can also express PD-1 [75], and therefore, they can be targeted by anti-PD-1 antibodies. Based on previously published data indicating that PD-1 and FoxP3 cooperate for the maintenance of fully functional T_regs_ [76], Kamada et al. explored how PD-1 blockade can influence T_reg_ behavior in the context of HPD [26]. By examining 14 paired (pre- and post-treatment) fresh tumor samples obtained from 2 HPD and 12 non-HPD gastric cancer patients, the authors found that after anti-PD-1 antibody treatment, the ratio between proliferating (Ki67^+^) effector T_regs_ (eT_regs_) and Ki67^+^ CD8^+^ T cells among the tumor-infiltrating lymphocyte (TIL) compartment remained stable in HPD patients, while it was reduced in the non-HPD group. These data led to the hypothesis that if the frequency of CD8^+^ lymphocytes does not overcome the frequency of eT_regs_, there is an increased possibility of developing HPD. A deeper analysis, conducted to better define eT_reg_ phenotype, revealed that PD-1^+^ eT_regs_ proliferated more than their PD-1^−^ counterparts and that PD-1^+^ eT_reg_ proliferation can be further increased by anti-PD-1 antibody treatment. In summary, blocking PD-1 on PD-1^+^ eT_regs_ enhanced their immunosuppressive ability. The observations made in the human setting were confirmed using a mouse model in which T_regs_ were made deficient in PD-1 expression. Knocking out PD-1 in T_regs_ resulted in greater proliferation paralleled by a more potent immunosuppressive capacity of these immune cells, resulting in the ability to boost tumor growth when transplanted into tumor-bearing mice [26].

Based on the previously described data, when we consider the possible mechanisms involving adaptive immune cells, it is reasonable to indicate T_regs_ as the prime suspects in the context of HPD. However, it should be taken into consideration that also non-T_regs_ CD4^+^ cells are not above suspicion.

It is certainly true that the role of non-T_regs_ CD4^+^ lymphocytes during ICI therapy has not been deeply investigated as for CD8^+^ cells, even if they exert fundamental functions, for instance initiating and sustaining the immune response, as recently reviewed in [77]. Therefore, as already described for other immune cells, it is likely to expect that, in particular circumstances, also non-T_regs_ lymphocytes can unexpectedly respond to ICI therapy, resulting in a detrimental outcome. Indeed, to this regards, it is possible to find some insights in the literature. For example, analyzing a cohort of 70 lung cancer patients treated with anti-PD-1 or anti-PD-L1 antibodies after progression to platinum-based chemotherapy, Arasanz and co-coworkers performed an immunophenotyping on peripheral blood samples collected at baseline and after the second cycle of treatment, focusing their attention on CD4^+^ lymphocytes. The obtained results were then correlated to patient clinical outcome. They found that HPD patients were characterized by a strong expansion of highly differentiated CD28^−^ CD4^+^ T cells, defined as CD4^+^ T_HD_, between the first and second cycle of therapy. Accordingly, the evaluation of tumor growth kinetics, higher in the HPD group, correlated with a greater presence of this CD4^+^ subpopulation. Although more detailed analyses are required to better characterize the phenotype of CD4^+^ T_HD_ lymphocytes, this work highlights the fact that also non-T_regs_ CD4^+^ cells may play an active role in triggering HPD [78].

Another important evidence about the possible involvement of non-T_regs_ CD4^+^ lymphocytes in determining HPD comes from the work by Zappasodi et al. The Authors observed, in a mouse model of melanoma, that a subpopulation of CD4^+^Foxp3^−^PD-1^high^, named 4PD-1^hi^, is present in the TME and that its frequency is correlated with the tumor size. Subsequent analyses revealed that 4PD-1^hi^ cells can exert immunosuppressive function by limiting proliferation, activation and pro-inflammatory cytokine production of CD4^+^ and CD8^+^ T cells, both in humans and mice. Although characterized by immunosuppressive properties, 4PD-1^hi^ T lymphocytes are distinct from T_regs_, since RNA-based sequencing expression profile and phenotypic analysis showed that they are more similar to CD4^+^ follicular helper T (T_FH_) cells. For the purpose of the present Review, the most intriguing data is that, while anti-PD-1 antibody treatment was able to reduce the number of these lymphocytes, anti-CTLA4 antibody administration increased their intra-tumor abundance, most probably due to an excessive T cell priming mediated by B lymphocytes [79]. Therefore, these data provide the demonstration that also non-T_regs_ CD4^+^ lymphocytes can unexpectedly respond to ICI therapy, in this specific case to CTLA-4 blockade, not only increasing their proliferation but also acquiring negative regulatory immune properties. Although the Authors considered these cells only a negative prognostic factor of ICI, it is undeniable that an augmented presence of these immunosuppressive cells may represent a fertile ground for HPD development.

While the role played by T lymphocytes and, in particular, by T_regs_ in the context of HPD is now well delineated, when considering B lymphocytes, it appears that B cells may not be involved in inducing the detrimental effect associated with ICI therapy. B cells can express PD-1, and it is now recognized that anti-PD-1 antibodies can enhance humoral immunity [80]; accordingly, some authors found the presence of B lymphocytes in the TME to be positively correlated with a favorable outcome to PD-1 blockade therapy [81, 82]. On the other hand, other researchers found that the absence of B cells does not have any impact on the efficacy of immunotherapy [83]. These discrepancies might be explained by the fact that there are many subsets of B cells, each of them with its peculiar functions [84–86]. Therefore, depending on the presence in the tumor bed of a particular B cell population, the final ICI therapy outcome can vary greatly. Although B lymphocytes can influence ICI therapy outcome, based on the available data, there is no evidence that these immune cells participate in determining HPD. A summary of the proposed adaptive-immune-mediated mechanisms is provided in Table 3.

**Table 3:**
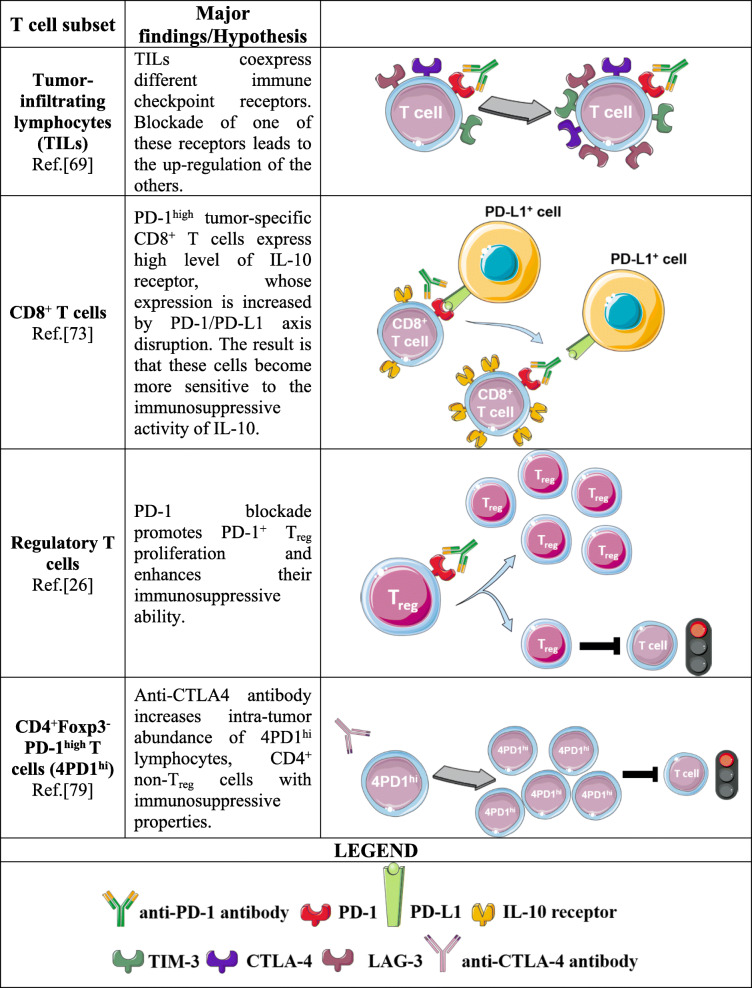
Possible immune-mediated mechanisms of HPD involving T cells

Possible role of T lymphocytes in determining HPD. PD-1 blockade increases the expression of other immune checkpoint receptors on tumor-infiltrating lymphocytes or determines the upregulation of IL-10 receptor on CD8^+^ T cells, making these cells more sensitive to IL-10-mediated immune suppression. Moreover, blocking PD-1 on T_regs_ exacerbates their immunosuppressive properties, while anti-CTLA4 antibody promotes the proliferation of CD4+Foxp3-PD-1^high^ T cells, lymphocytes with immunosuppressive properties

### Role of the fc domain

The publications reported so far describe the effects of the binding between ICIs and their own specific antigens (i.e., PD-1 or PD-L1). However, an antibody is composed of two functionally distinct domains: the F (ab’)_2_ fragment that binds the antigen and the fragment crystallizable (Fc) region that is recognized by membrane molecules expressed by several immune cells, named immunoglobulin Fc receptors (FcRs) [87, 88]. The interaction between the Fc domain of an antibody and its cognate FcR triggers a cascade of immune events stimulating phagocytic or cytotoxic cells to eradicate pathogens or infected/tumor cells through different mechanisms, such as antibody-dependent cell-mediated cytotoxicity (ADCC), complement-dependent cytotoxicity (CDC) and antibody-mediated phagocytosis (ADCP) [87, 89]. To avoid the killing of T cells that express ICs, most ICIs were developed by choosing antibody isotypes with low or significantly reduced binding to FcRs [90–92]. Indeed, nivolumab and pembrolizumab, anti-PD-1 antibodies, are IgG4, an isotype considered immunologically inert but that still retains the ability to bind FcγRI and FcγRIIb, activating and inhibitory FcRs, respectively [90–92]. In this regard, it has been reported that tumor-associated macrophages (TAMs) are able to capture anti-PD-1 antibodies from the T cell membrane through FcγRIIb [93].

Although it is not known whether this interaction may have biological consequences, our work provided some important insights [29]. Analyzing NSCLC tumor specimens collected before the initiation of ICI immunotherapy, we observed the presence of peculiar clusters of TAMs characterized by epithelioid morphology and CD163, CD33 and PD-L1 coexpression in all HPD patients, suggesting the involvement of macrophages in HPD occurrence. To investigate whether these innate immune cells could play a role in inducing HPD, in vivo experiments using different NSCLC immunodeficient mouse models were performed. We were able to reproduce HPD-like progression in mice after anti-PD-1 antibody administration. Subsequent analyses suggested that, as in cancer patients, a particular macrophage population present in the TME before starting anti-PD-1 antibody therapy might be the immune cell responsible for the development of HPD through a mechanism that does not involve the direct blocking of PD-1 on these immune cells. Therefore, we speculated that the detrimental boost in tumor growth may be ascribed to the Fc domain of the antibody. Indeed, the use of the anti-PD-1 antibody F (ab’)_2_ fragment as well as macrophage depletion completely abrogated the marked increase in tumor growth observed with the whole antibody. Since anti-PD-1 antibody is able to bind the inhibitory receptor FcγRIIb, we imagined a scenario in which a particular subset of TAMs expressing the inhibitory receptor FcγRIIb undergoes functional reprogramming after interaction with the Fc domain of the anti-PD-1 antibody acquiring enhanced pro-tumor properties, eventually inducing HPD [29].

Another explanation regarding the possible role FcγRIIb in the context of HPD came from the comment made by Knorr and Ravetch to [29]. Based on prior studies describing that the in vivo activity of some therapeutic antibodies is dependent on FcγRIIb expression in the TME, the Authors described a situation in which anti-PD-1 antibody creates a type of bridge between FcγRIIb^+^ immune cells and PD-1^+^ macrophages. This interaction may be able to induce the clustering of several PD-1 molecules on macrophage membranes. The close proximity of different PD-1 proteins may trigger PD-1-mediated signaling, leading to macrophage polarization towards the pro-tumor phenotype [94].

Although FcγRIIb is potentially responsible for HPD through at least two distinct mechanisms, the involvement of other FcRs cannot be excluded. For instance, Swisher et al. showed that IgG4 immune complexes (ICs) were able to limit the response to IFN-γ in human monocyte-derived macrophages through the FcγRI interaction. Moreover, the activation of FcγRI mediated by IgG4 ICs polarized monocyte-derived macrophages towards the M2-like phenotype [95]. The Fc-mediated mechanisms of HPD are summarized in Table 4.

**Table 4:**
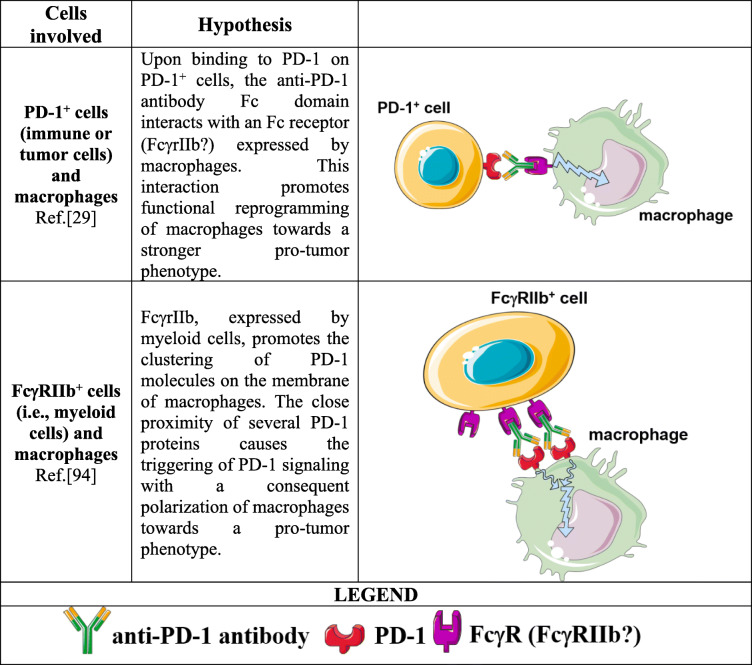
Possible mechanisms of HPD involving the interaction between the ICI Fc domain and macrophage Fc receptors

The engagement of an unidentified Fc receptor expressed by TAMs by the anti-PD-1 antibody Fc domain may directly trigger a functional reprogramming of these immune cells or may induce the clustering of PD-1 on macrophages, leading, in both cases, to the acquisition of an enhanced pro-tumor phenotype

## Possible tumor-related mechanisms of HPD

Considering that HPD consists of a rapid increase in tumor growth after ICI administration, this marked acceleration of the disease may not be a mere reflection of an unexpected response of the immune system to ICI treatment, but it is possible that intrinsic characteristics of tumor cells may play an active role in determining this clinical phenomenon. Indeed, many efforts have focused on understanding whether particular tumor genomic alterations are responsible for HPD occurrence to find a biomarker suitable to predict HPD. However, the majority of the published studies only describe an association between the tumor mutational landscape and HPD, and very rarely, a possible mechanistic explanation has been provided.

### *MDM2/MDM4* amplification and *EGFR* alteration

The first genomic alterations that were associated with HPD are *MDM2/MDM4* amplification and *EGFR* mutation [31]. The biological function of *MDM2* (murine double minute 2) and its homolog *MDM4* is to negatively regulate the tumor suppressor p53 by promoting its proteasome degradation [96]. By analyzing 155 stage IV patients affected by different types of cancers using next-generation sequencing (NGS), Kato et al. observed that in a cohort of 155 patients with advanced cancers, 49 (31.6%) had a TTF < 2 months; 6 of them had *MDM2/MDM4* amplifications that were found to be an independent predictor of poor clinical outcome by multivariate analysis. Among these 6 patients, 4 were identified as hyperprogressors. Subsequently, this finding was also supported by several other studies [97–99]. Although the mechanism by which *MDM2/MDM4* amplifications could induce HPD is unknown, the Authors hypothesized that the increase in IFN-γ levels in the TME following ICI administration [100] may trigger JAK/STAT signaling [101] in tumor cells, resulting in an upregulation of the interferon regulatory factor (*IRF*)-*8* gene [102]. IRF8 is known to bind the *MDM2* promoter, favoring its expression [103]. Therefore, an augmented MDM2 level may determine a stronger p53 inhibition leading to a dysregulation of cancer cell proliferation. However, the relevance of *MDM2* in affecting ICI therapy outcome has been highlighted by the work of Fang et al. To investigate how p53 can influence the response to anti-PD-1 immunotherapy, the authors evaluated the ability of the MDM2 inhibitor APG-115, currently under clinical investigation (ClinicalTrials.gov Identifier: NCT02935907), to improve the effect of anti-PD-1 treatment. In vitro and in vivo studies have shown that APG-115 can modulate the immune response by repolarizing protumor M2 macrophages towards an M1 phenotype, enhancing T cell activation and upregulating PD-L1 expression on tumor cells. Finally, APG-115 was able to enhance PD-1 blockade antitumor activity [104]. Since it has been reported that MDM2 can modulate anti-PD-1 antibody efficacy, its involvement in HPD onset cannot be excluded.

In addition to *MDM2/MDM4* amplifications, the analysis provided by Kato et al. revealed that 2 out of 10 patients with *EGFR* alterations experienced HPD. Accordingly, other studies reported similar observations. EGFR was the first member of the ErbB family to be discovered, and it plays a significant role in many cellular processes essential for survival and cell growth. EGFR is also known to be involved in the pathogenesis and progression of different types of cancers [105] and in promoting immune escape through different mechanisms, such as the upregulation of PD-L1, the downregulation of tumor antigen presentation, and the induction of secretion of metabolites and molecules with immunosuppressive properties in the TME [106]. Therefore, mutations in this receptor may be associated with nonresponse to ICI therapy [107, 108], but the possible link between EGFR and HPD remains unknown.

### PD-L1 and VEGFR2 polymorphisms

Other possible HPD-related genomic alterations were identified by Refae and coworkers [109]. The Authors evaluated the frequency of 17 SNPs in 4 genes (*PD-1*, *PD-L1*, *IDO1* and *VEGFR2*), selected for their functional and clinical relevance, in a cohort of 98 patients, affected by different types of cancer and treated with anti-PD-1 or anti-PD-L1 monotherapy alone. In this cohort, 14% of patients experienced HPD during therapy. Multivariate analysis revealed that the polymorphisms rs2282055 G allele in the *PD-L1* gene and rs1870377 A allele in the *VEGFR2* gene are significantly and independently associated with a higher susceptibility to developing HPD. Based on the data available in the GTEX portal, the Authors hypothesized that rs2282055 *PD-L1* polymorphisms may have a role in regulating the PD-L1 expression level that, in turn, may impact ICI therapy outcome, even though the real influence of PD-L1 expression on ICI treatment is still a matter of debate [110]. In the case of the *VEGFR2* gene, which encodes a receptor that responds to the VEGF signal and regulates endothelial migration and proliferation [111], rs1870377 is reported to increase the affinity and activity of VEGF-A on VEGFR2 with the result of promoting angiogenesis, as reported in nonsmall cell lung cancer tissues [112]. Moreover, VEGFR2 can boost cancer proliferation and metastasis independently of proangiogenic stimuli [113]. Although these data are intriguing, they do not provide a biological explanation about how these polymorphisms and anti-PD-1/PD-L1 antibodies can work together in determining HPD. However, we can speculate that if ICIs are able to induce a stronger M2 phenotype in TAMs in some particular circumstances, as described in the previous section, one of the features of M2 macrophages is the ability to release VEGF that may more efficiently trigger the signaling pathways mediated by VEGFR2, exploiting the increased affinity of this receptor determined by the rs1870377 polymorphisms. Of note, this work highlighted the possible existence of a tight link between angiogenesis and HPD, and in this regard, it is worth mentioning the ability of ICI to induce the expression of pro-angiogenic molecules. For example, Wu et al. reported an increased serum level of angiopoietin-2 (ANGPT2), a molecule involved in blood vessel maturation with potential protumor activity [114], in patients with advanced melanoma who progressed after ICI treatment [115]. The biological activity of ANGPT2 is not only restricted to neoangiogenesis but can also impact the macrophage phenotype by promoting PD-L1 expression [115] and IL-10 secretion [116], which, in turn, are involved in the expansion of CD4^+^CD25^high^Foxp3^+^ Tregs in tumor-bearing mice [116]. These data suggest that ICI-induced angiogenesis might occur to create a stronger immunosuppressive microenvironment. Moreover, the augmented tumor growth kinetics presume higher energy consumption by tumor cells, and new vasculature formation may provide the nutrients required to sustain such rapid cell proliferation. Furthermore, HPD is often characterized by the appearance of metastatic foci, and more blood vessels vascularizing the tumor bed may represent an escape route for cancer cells, increasing the possibility for dissemination throughout the body.

### Alterations of *KRAS*, *FBXW7* and *STK11*

Sasaki et al. performed a retrospective study on 64 advanced gastric cancer (AGC) patients treated with nivolumab [117]. Among 13 patients who developed HPD after treatment, three showed *FBXW7* mutation, a tumor suppressor gene encoding a protein belonging to the proteasome systems [118], and three others showed amplification in the *KRAS* gene, a GTPase that regulates several cellular signaling pathways [119]. They also observed that absolute neutrophil count (ANC) and C-reactive protein (CRP) level significantly increased only in patients with HPD during the first months of nivolumab treatment. Although the Authors speculated that higher ANC and CRP levels in HPD patients may be correlated with a strong release of neutrophils from bone marrow and a consequent accumulation of myeloid-derived suppressor cells (MDSCs) in tumors, the link with the reported genomic abnormalities was not explored.

Genomic alterations of the *KRAS* gene were also found in HPD patients by Kim et al. in a retrospective study of 335 patients with advanced NSCLC treated with anti-PD-1 or anti-PD-L1 [25]. They found that 3 out of 16 patients with HPD had *RAS*/*RAF* mutations (two *KRAS* and one *RAF* mutation) and, at the same time, serine/threonine kinase 11 gene (*STK11*) truncating mutations, another tumor suppressor gene with a broad spectrum of functions ranging from cellular metabolism to regulation of apoptosis [120]. Based on a previously published work [72], the Authors speculated that the concomitant loss of *STK11* together with mutations in an oncogenic pathway (*KRAS/RAF*) promotes IL-6 secretion by tumor cells, which, in turn, can determine the recruitment of neutrophils in the tumor microenvironment.

Since a consistent infiltration of neutrophils can be associated with worse outcome to ICIs [121], one of the simplest method to evaluate their presence is the so-called neutrophil to lymphocyte ratio (NLR). NLR is calculated by dividing the number of neutrophils by the number of lymphocytes. These numbers are usually obtained from peripheral blood sample analysis, NLR represents a useful parameter utilized to evaluate the general immune response and systemic inflammatory status [122–124]. In the context of ICI, NLR demonstrated a very good clinical utility. For example, Mezquita et al. analyzed the neutrophils/(leukocytes - neutrophils) ratio (dNLR), in a cohort of NSCLC patients treated with ICIs or standard chemotherapy. The found that high dNLR (high number of circulating neutrophils) was independently associated with poor outcome in the ICIs but not in the chemotherapy group [125]. Similar results were also obtained in metastatic urothelial cancer where, again, NLR was significantly predictive of patients with progressive disease following ICIs [126]. Moreover, in a recently published conference proceeding, Ferrara et al. described a correlation between tumor and circulating neutrophils and HPD. The Authors reported the at the baseline, before the initiation of ICI treatment, the percentage of circulating immature low-density neutrophils, identified as CD10^−^ CD15^+^ CD66b^+^ cells, was significantly higher in HPD versus non-HPD patients. In addition, a subpopulation of Gr1^high^Ly6C^low^IL-17^+^ neutrophils was found in mice treated with anti-PD-1 antibody that experience a HPD-like increase in tumor growth [127].

These results suggest that neutrophils can be considered as another important factor able to promote HPD, since an increased number of neutrophils at the tumor site may affect T cell number and function, exacerbating their exhaustion, resulting in a strong impairment of ICI activity.

### Tumor cell-intrinsic PD-1 expression

PD-1 is expressed by adaptive and innate immune cells. However, it is emerging that this protein can also be present on a small fraction of tumor cells, but its exact function remains poorly understood. The main question about PD-1 expression on tumor cells is to determine whether this molecule can act as a tumor suppressor or can exert protumor activity.

Using the PD-1-expressing mouse lung cancer cell line M109, Du et al. found that PD-1 overexpression or treatment with recombinant PD-L1 decreased cell viability. Conversely, in vitro PD-1 blockade or silencing had the opposite effect. Moreover, in vivo PD-1 blockade accelerated M109 tumor growth [128]. Based on the obtained data, a model in which PD-1 function is similar to that described for T lymphocytes was proposed. The engagement of PD-1 by its cognate ligand PD-L1 triggers an inhibitory signaling cascade able to negatively impact tumor proliferation. When the PD-1/PD-L1 axis is disrupted by antibody treatment, these inhibitory signals are abolished, and cell proliferation can be reactivated. Notably, both the M109 cell line and tumor tissue from NSCLC patients express both PD-1 and PD-L1; therefore, the PD-1/PD-L1 interaction may occur *in cis* in the same tumor cells. The authors speculated that HPD may be the sum of several independent events. For example, if the tumor is highly infiltrated by PD-1^+^ T lymphocytes, whose activity is dampened by the presence of PD-L1 on tumor and immunosuppressive cells, PD-1 blockade can restore T cell-based antitumor immunity. On the other hand, in a poorly infiltrating tumor, the majority of the available PD-1 molecules are those expressed by tumor cells that, if bound by a specific antibody, can promote tumor growth. Similar results were also obtained by Wang et al., who demonstrated that PD-1 inhibitory activity in tumor cells relies on AKT and ERK1/2 signaling pathways [129]. However, other studies, performed using melanoma and hepatocellular carcinoma models, described a completely different situation in which blocking PD-1 directly on cancer cells determined a marked reduction in tumor proliferation [130, 131]. Collectively, these data suggest that tumor cell-intrinsic PD-1 can oversee different biological processes depending on the tumor type, and therefore, its inhibition can lead to opposite outcomes.

Moreover, it is possible to extend the role of PD-1 in tumor cells based on the findings described in [29]. Using NSCLC patient-derived xenografts (PDXs) treated with the anti-human PD-1 antibody nivolumab, the authors were able to exclude the involvement of mouse PD-1, since nivolumab only binds human PD-1 and not its mouse homolog. Therefore, in PDX models, the only source of human PD-1 is represented by tumor cells. As described before, an increase in tumor growth after nivolumab treatment was observed in some PDXs, a phenomenon that was completely abolished using nivolumab F (ab)_2_ fragments or macrophage depletion. The Authors speculated that binding of anti-PD-1 antibody on tumor cells allows the Fc domain of the antibody to be in the right conformation to be recognized by FcRs expressed by macrophages. The possible consequences of this interaction have been discussed in the previous section. A summary of the proposed tumor-mediated mechanisms is provided in Table 5.

**Table 5:**
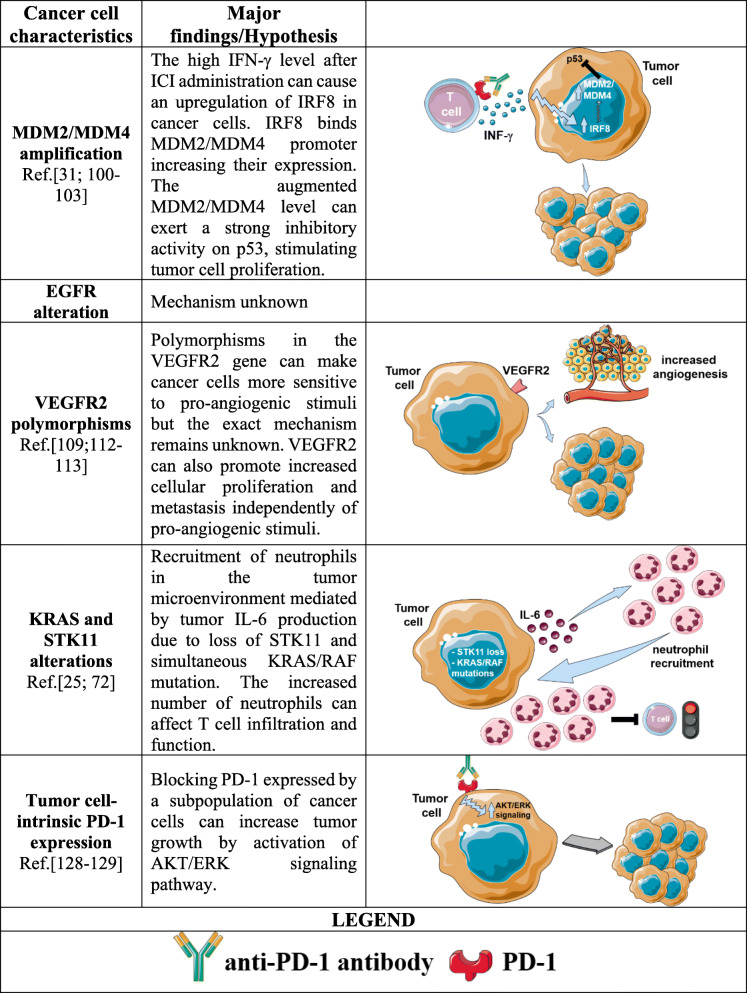
Possible tumor-mediated mechanisms of HPD

Peculiar genomic abnormalities in cancer cells as key determinants in triggering HPD upon ICI administration

### Chemotherapy: an old solution to a new problem?

**Fig. 1:**
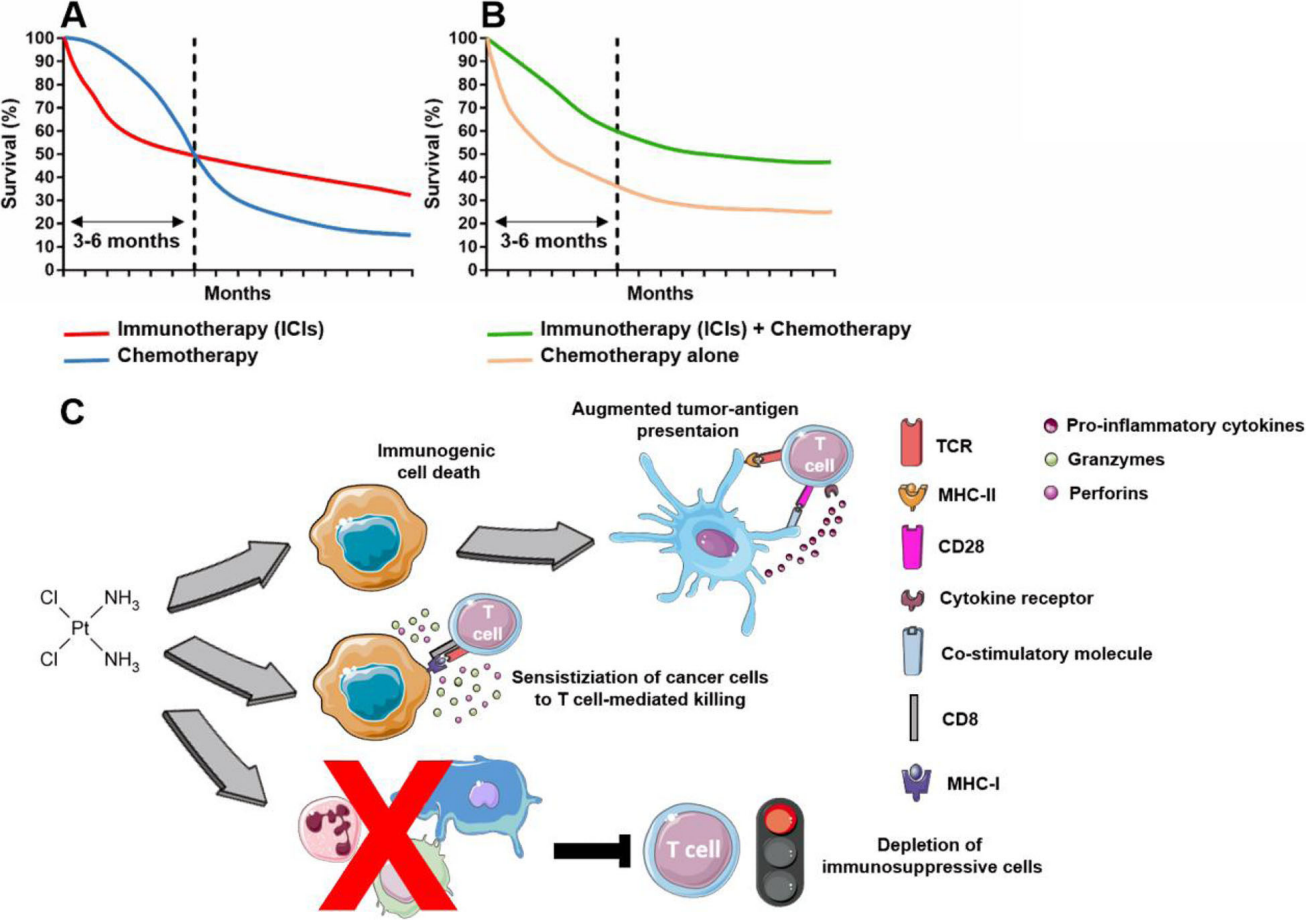
Effect of chemotherapy on HPD. **a** Representative image of the crossover of the survival curves observed in clinical trials comparing ICIs versus chemotherapy. **b** Representative image of survival curves observed in clinical trials comparing chemotherapy+ICIs versus chemotherapy alone. **c** Overview of the main immunological effect of platinum compounds

The survival analysis of four important phase III clinical trials, conducted to evaluate the efficacy of ICI therapy in comparison to standard chemotherapy (CheckMate 057, CheckMate 141, Keynote 0456, IMvigor2117) [5, 12, 132, 133], revealed an increased rate of progression and deaths in the immunotherapy arm compared to chemotherapy arm in the first three-six months after the initiation of the therapy, as clearly showed by the crossover of the survival curves (Fig. 1a). This was the starting point that induced clinicians and scientists to interrogate about a possible negative effect associated with ICI therapy. Subsequent investigations led to the identification of a novel pattern of progression that was defined hyperprogression.

Although ICI demonstrated good clinical activity, data from human studies indicated that only a fraction of patients could really benefit of this type of therapy, that, unfortunately, in the majority of cases resulted largely ineffective or, as described above, even detrimental [134]. The current view is that tumors that are characterized by an inflamed/immunogenic phenotype, the so-called “hot” tumors, have the highest chance to properly respond to ICI. On the contrary, cancers that lack infiltration of effector cells, named “cold” tumors, can rarely be responsive to ICI. In order to revert these tumors from immune-deprived to inflamed, combination strategies with other immunotherapeutic drugs or chemotherapy, have been evaluated [135].

One of the pioneering study in this field was the Keynote 021 (NCT02039674), a randomized, open-label, phase II clinical trial conducted on chemotherapy-naive, stage IIIB or IV, non-squamous NSCLC without targetable EGFR or ALK genetic alterations. Patients received carboplatium plus premetrexed in combination or not with the anti-PD-1 antibody pembrolizumab. The obtained results demonstrated that the addition of ICIs to the chemotherapeutic regimen was well tolerated and that progression-free survival was significantly longer in the pembrolizumab plus chemotherapy group compared to chemotherapy arm. When considering the survival analysis, it emerges the lack of the already mentioned crossing of the two curves observed in the first three-six months of the clinical trials with ICIs as monotherapy (Fig. 1b). In this case, the immunotherapy plus chemotherapy cohort demonstrated a superior anti-tumor effects for the entire duration of the follow-up [136]. Subsequent studies confirmed these initial findings [137], as also clearly shown in a recently published meta-analysis [138]. Indeed, Addeo et al. collected the data from several randomized clinical trials in which chemotherapy was administered with ICIs as first-line treatment for metastatic NSCLC patients. The Authors compared the PFS and OS of the chemotherapy alone group versus the chemotherapy plus immunotherapy arm. They found that in all considered clinical trials the combination of chemo/immunotherapy conferred an advantage in terms of OS and PFS compared to chemotherapy alone [138]. Apparently, no signs of HPD were detected in these trials. Taken together, these results suggest that adding a chemotherapeutic regimen to ICIs is a potential strategy to avoid the onset of HPD.

Although a certain mechanistic explanation of this phenomenon has not been provided yet and needs to be fully clarified, it is always possible to make some speculations based on what has been already reported in the literature. It is a long-held belief that chemotherapeutic drugs mainly exert a negative effect on the immune system, by killing effector cells or by suppressing/dampening their function. However, it is now well known that most of cytotoxic drugs have the ability to positively boost the immune response and that their immune-stimulating ability represents an important component of their overall anti-tumor activity. Obviously, each drug may elicit the immune response in different ways, such as making cancer cells more recognizable for the immune attack (i.e. increased APCs maturation and antigen presentation), depleting immunosuppressive cells or directly stimulating cells with tumor-killing activity [139, 140].

In the majority of the clinical trials considered by Addeo et al. [138], the chemotherapeutic drugs utilized are platinum or its derivatives that belong to the alkylating agents, a class of drugs able to cross-link DNA strands, thereby inhibiting DNA synthesis, eventually inducing the apoptosis of cancer cells. Beyond their cytotoxic activity, this type of chemotherapy also profoundly impacts on the immune TME. After been damaged by chemotherapy, tumor cells start to expose calreticulin on their plasma membrane that, acting as “eat-me” signal, favors the phagocytosis of dying cells and cellular debris by APCs, in particular DCs. It should be noted that platinum does not induce calreticulin exposure, while some of its derivatives do. Moreover, platinum compounds induce neoplastic cell to release ATP, a molecule able to induce DCs recruitment and activation/maturation, and High Mobility Group Box 1 (HMGB1) that triggers TLR4 on DCs leading to an enhanced activation and antigen presentation capacity. This type of chemotherapy can also directly act on DCs attenuating STAT6 signaling and determining a downregulation of PD-L2, thus reducing the immunosuppressive potential of these immune cells. All these mechanisms, that can be grouped together under the same cellular process defined “immunologic cell death” (ICD), concur to promote a more efficient and stronger tumor-antigen presentation to T cells [141–143]. In addition to ICD, platinum drugs can also sensitize cancer cells to T cell attack: first, making these cells more sensitive to granzyme-B-mediated killing and, second, by increasing MHC class I expression. Finally, some platinum derivatives, such as oxaliplatin, are able to reduce the number of MDSCs and their immunosuppressive properties [141–143] (Fig. 1c).

Collectively, these results suggest that even if a tumor is characterized by the presence of immune cells that potentially can trigger HPD upon ICIs treatment, the concomitant administration of standard chemotherapy should eliminate or, at least, constrain one or more factors able to induce HPD. Moreover, the ability of chemotherapy to abrogate HPD will be an extremely useful tool for scientists for better understanding HPD.

### HPD or not HPD: that is the question

**Fig. 2:**
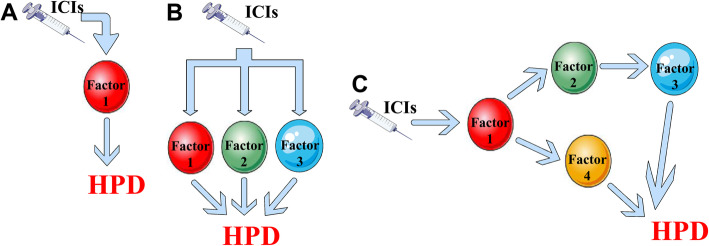
Different possible models of HPD development. **a** HPD as a monofactorial process. **b** HPD as a multifactorial process. **c** HPD as a cascade of simultaneous events

It is evident that HPD, at present, remains a quite elusive clinical problem. In this Review, we tried to summarize not only the mechanisms that are already demonstrated to be a cause of HPD but also those that we considered to be possibly linked to this phenomenon. Every single study discussed here represents a piece that fits in with a larger picture. However, since the study aimed at identifying the cellular and molecular origin of HPD is still at its infancy and the data at our disposition are very few, we cannot say for sure if only one of the proposed mechanisms can be sufficient to trigger HPD (Fig. 2a) or whether this detrimental outcome to ICI therapy requires the coexistence of two or more contributing factors (Fig. 2b). It is also possible to imagine a scenario in which the presence of one of the above-cited factors may constitute a *conditio* *sine qua non* for HPD development, initiating after interaction with ICIs a cascade of events able to negatively affect all the surrounding tumor microenvironment and promoting tumor growth, in a sort of domino effect (Fig. 2c). Therefore, a single factor might be not able *per se* to induce HPD but might need to be a part of an intricate network of concurrent causes and, based on the nature of this combination, it may trigger HPD or not.

Moreover, if we analyze what has been discussed before, we can notice that all the described mechanisms fall under two big groups: those promoting an exacerbation of the immune suppression, and those conferring to cancer cells an advantage in terms of survival/proliferation. However, these are some of those mechanisms that might increase tumor aggressiveness without any involvement of HPD. Therefore, one may rightly ask (i) whether these causative factors can be at the basis of both progression of the disease (PD) and HPD and (ii) if and how it is possible to discriminate between those causing the former or the latter. We believe that the central question is: “What is HPD?”. This only partially answered question represents the central problem. To date, there is no consensus about the definition of HPD and, the dividing line between PD and HPD can be very thin and variable, depending on the criteria utilized for their definition. The comparison of data obtained from different research groups can be extremely challenging since the results may be influenced by the different classification systems. Moreover, as already said, if HPD is a multifactorial process, it might be very likely that a single mechanism may induce PD and only the combination of all those mechanisms together, like actors in the same play, might fully elicit HPD. However, based on what found in the literature, it is possible to make some speculations about how different mechanisms could be considered particularly related to HPD rather than in common with PD. Table 6 summarizes this attempt.

**Table 6:**
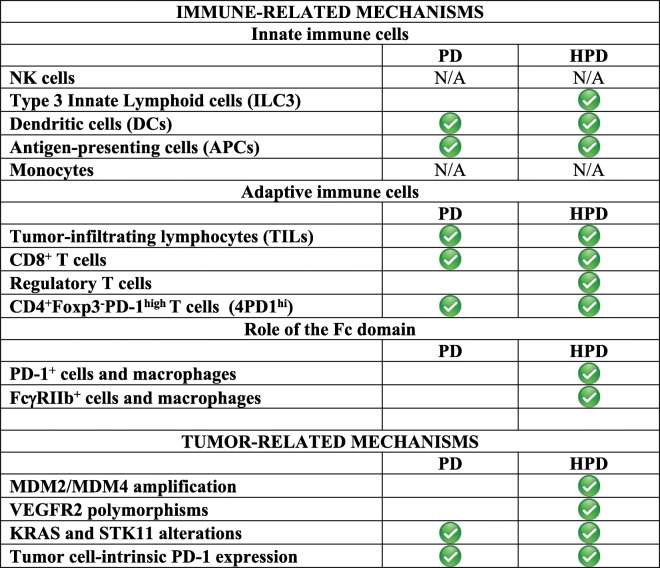
Comparison between mechanisms only related to HPD or shared with PD

*HPD* Hyperprogressive disease, *PD* Progressive disease, *N/A* Not assigned

## Conclusions

Since it cannot be denied that tumor progression unexpectedly speeds up in a fraction of patients during ICI immunotherapy, the understanding of this provocative phenomenon still remains an important challenge. The investigations aimed at elucidating the causes of HPD have important translational relevance and applications. Indeed, a better understanding of the mechanisms triggering HPD could lead to the identification of patients not to be treated with ICIs and to the development of novel approaches able to reverse this extremely negative clinical phenomenon.

## Data Availability

Not applicable.

## Abbreviations

ICIs: Immune checkpoint inhibitors
CTLA-4: Cytotoxic T lymphocyte-associated protein 4
PD-1: Programmed cell death protein 1
PD-L1/L2: Programmed cell death ligand 1 and 2
TME: Tumor microenvironment
JAK1/2: Janus kinases 1 and 2
HPD: Hyperprogressive disease
NSCLC: Non-small cell lung cancer
HNSCC: Head and Neck squamous cell carcinoma
AGC: Advanced gastric cancer
TGR: Tumor growth rate
TGK: Tumor growth kinetics
TTF: Time-to-treatment failure
ILCs: Innate lymphoid cells
DCs: Dendritic cells
TIDCs: Tumor-infiltrating dendritic cells
TAMs: Tumor-associated macrophages
FcRs: Immunoglobulin Fc receptors
MDM2/4: Murine double minute 2/4
IRF-8: Interferon regulatory factor 8
EGFR: Epidermal growth factor receptor
VEGFR2: Vascular endothelial growth factor receptor 2
ANGPT2: Angiopoietin-2
ANC: Absolute neutrophil count
STK11: Serine/threonine kinase 11
